# Computational elucidation of stomidazolone mediated inhibition of stomatal differentiation and its implication in plant developmental regulation

**DOI:** 10.1371/journal.pone.0329401

**Published:** 2026-02-10

**Authors:** Syeda Sumayya Tariq, Urooj Qureshi, Mamona Mushtaq, Maria Nasim, Mohammad Nur-e-Alam, Tawfeq A. AlHowiriny, Yan Wang, Zaheer Ul-Haq

**Affiliations:** 1 Dr. Panjwani Center for Molecular Medicine and Drug Research, International Center for Chemical and Biological Sciences, University of Karachi, Karachi, Pakistan; 2 H.E.J. Research Institute of Chemistry, International Center for Chemical and Biological Sciences, University of Karachi, Karachi, Pakistan; 3 Department of Pharmacognosy, College of Pharmacy, King Saud University, Riyadh, Kingdom of Saudi Arabia; 4 State Key Laboratory for Chemistry and Molecular Engineering of Medicinal Resources, School of Chemistry and Pharmaceutical Sciences, Guangxi Normal University, Guilin, China; Kwara State University, NIGERIA

## Abstract

Stomata play a critical role in plant physiology by balancing gas exchange and water conservation. Their development is driven by a precisely orchestrated sequence of cell divisions and differentiation events, regulated by basic helix-loop-helix (bHLH) transcription factors such as MUTE. Previous research reports stomidazolone, a doubly sulfonylated imidazolone derivative, as an effective inhibitor of stomatal development which has been shown to bind strongly to MUTE, interfering with its interaction with SCRM, effectively suppressing stomatal differentiation. The ACTL domain, a conserved structural feature in plant bHLH proteins, acts as a potential site for chemical inhibition, enabling selective disruption of stomatal formation. This suggests a promising approach for enhancing drought resilience in plants by reducing water loss through transpiration. While experimental data support stomidazolone’s inhibitory role, the molecular details of its binding to MUTE remain inadequately characterized. To address this gap, a comprehensive in silico analysis combining molecular docking and density functional theory (DFT) was performed to elucidate the binding interactions, electronic properties, and reactive potential of stomidazolone, thereby uncovering the molecular features that underpin its affinity and specificity toward MUTE. An all-atom molecular dynamics (MD) simulations was then carried out to provide mechanistic insights beyond static binding models, followed by a number of post-simulation analyses assessing system stability and dynamics to gain deeper insight into the Stomidazolone-Mediated Inhibition of MUTE. Our results reveal the formation of a stable and compact stomidazolone–MUTE complex, characterized by a lower average RMSD (1.45 ± 0.12 nm) compared to the Apo state (1.65 ± 0.18 nm), while hydrogen bonding analysis further demonstrated persistent interactions involving Arg62 and Ser69, along with a stabilizing contribution from Glu163, collectively supporting the strong binding affinity of stomidazolone within the MUTE active site. Principal component analysis further highlighted the conformational coherence and coordinated atomic motion, while the free energy landscape showed well-defined energy minima, underscoring the stability of the interaction and energetic favorability of the complex. Together, these findings provide a molecular framework for understanding the inhibitory mechanism of stomidazolone on MUTE, offering a basis for the rational design of next-generation agrochemicals targeting stomatal development. The study also highlights the conceptual novelty of small-molecule modulation of lineage-specific transcription factors as a potential strategy for the synthetic control of plant developmental plasticity. While the results are computational, they outline clear directions for experimental validation and scaffold optimization, paving the way for future efforts to translate these insights into practical applications for improving crop resilience and water-use efficiency. Importantly, this study provides the first mechanistic, residue-level insight into how stomidazolone engages the ACT-Like (ACTL) domain of MUTE, revealing the specific molecular interactions and dynamic features that underpin its inhibitory effect.

## 1. Introduction

Plant growth and survival rely on stomata (microscopic, adjustable valves on the shoot epidermis) which is mainly responsible for regulating gas exchange while minimizing water loss. Stomatal movement and development are tightly controlled by environmental factors such as light availability, drought stress, and atmospheric CO₂ levels, all of which influence the plant’s water-use efficiency [1]. The formation of stomata follows a highly coordinated sequence of cell divisions and differentiation events during leaf development, orchestrated by a network of basic-helix-loop-helix (bHLH) transcription factors (TFs) [2,3]. Among these, SPEECHLESS (SPCH), MUTE, and FAMA play pivotal roles, each driving specific cell-state transitions. These factors function through heterodimerization with their bHLH partners SCREAM (SCRM and SCRM2), forming transcriptional complexes that ensure proper stomatal lineage progression [4,5].

A particularly intriguing feature of these bHLH transcription factors is the presence of a C-terminal ACT-Like (ACTL) domain, a structurally conserved module resembling the ACT domain found in various metabolic enzymes across bacteria and eukaryotes [6]. Recent studies have revealed that the ACTL domain of SCRM serves as a selective interface for heterodimerization with SPCH, MUTE, and FAMA [7]. This discovery suggests the ACTL domain as a potential target for chemical inhibition, providing a strategy for selectively perturbing stomatal differentiation. While the ACTL domain is widely conserved among plant bHLH proteins, no known small-molecule inhibitors targeting this domain or bHLH-ACTL transcription factors in general have been identified [8,9].

Over the past decade, both experimental and computational studies have progressively advanced our understanding of stomatal regulation and the functional role of ACT-like (ACTL) domains in plant bHLH transcription factors. Experimental work in Arabidopsis thaliana has elucidated the hierarchical regulatory cascade involving SPCH, MUTE, and FAMA, which orchestrates stomatal lineage progression, with MAP kinase signaling directly modulating SPCH activity [10]. Structural and phylogenetic analyses have further revealed that approximately one-third of plant bHLH families possess a conserved C-terminal ACTL domain, which influences dimerization specificity and regulatory behavior, suggesting a potential ligandable interface [11]. More recently, genetic and biochemical studies suggest that the ACTL domain of SCREAM (SCRM) functions as a selective interaction interface, determining partner specificity among SPCH, MUTE, and FAMA [12].

Although previous chemical screens have identified compounds capable of suppressing stomatal movement, they have not been designed to selectively disrupt protein interactions governing stomatal differentiation [13]. Notably, Nakagawa et al. reported the discovery of stomidazolone, a doubly sulfonylated imidazolone compound that serves as a potent inhibitor of stomatal differentiation. Through both in vivo and in vitro experiments, stomidazolone was shown to bind tightly to MUTE, thereby disrupting its interaction with SCRM. This inhibition of heterodimerization effectively arrests stomatal differentiation, reducing stomatal density [14]. By limiting water loss through transpiration, such an approach could offer a promising strategy for enhancing plant resilience in arid conditions while preserving growth potential.

While experimental data support stomidazolone’s inhibitory role, the molecular details of its binding to MUTE remain inadequately characterized. To address this gap, the present study undertakes an in-depth in silico investigation of the molecular interactions between stomidazolone and the MUTE ACT-Like (ACTL) domain, integrating complementary computational techniques to derive mechanistic insights. Molecular docking was first employed to identify the most favorable binding sites and key interacting residues, providing an initial understanding of stomidazolone’s recognition pattern within the MUTE binding pocket. Density Functional Theory (DFT) calculations further refined these findings by optimizing the geometry of stomidazolone and characterizing its electronic structure, reactivity, and charge distribution, which underpin its potential for specific molecular interactions. Subsequently, all-atom Molecular Dynamics (MD) simulations were performed over 300 ns to probe the structural stability, flexibility, and dynamic behavior of MUTE in both its Apo and complexed states, revealing how ligand binding influences protein motion and compactness over time. To extract deeper insights from these trajectories, Principal Component Analysis (PCA) was used to delineate the dominant modes of collective motion, while Free Energy Landscape (FEL) mapping captured the thermodynamic stability and conformational preferences of the system, thereby illustrating the energetic favorability of complex formation. By providing detailed insights into the molecular basis of stomidazolone’s binding to the MUTE ACTL domain, this study aims to provide the groundwork for targeted chemical modulation of ACTL-containing plant bHLH transcription factors.

Such selective perturbation strategies hold significant potential for the transient manipulation of plant development, offering novel avenues for improving water-use efficiency and crop resilience in response to environmental challenges. While reduced stomatal density can contribute to improved water-use efficiency and potentially enhance plant resilience under arid conditions, this adaptation may also entail trade-offs, such as limited CO₂ uptake and reduced photosynthetic capacity. Therefore, any chemical or genetic modulation of stomatal development should aim to achieve a balanced regulation that minimizes water loss without compromising carbon assimilation or overall plant productivity.

## 2. Methodology

### 2.1 Ligand optimization using Density Functional Theory (DFT)

To gain insight the molecular geometry optimization and single point energy of the stomidazolone were carried out by the CP2K program [15]. The hybrid DFT method was executed using the Becke, 3-parameter, Lee Yang-Parr (B3LYP) exchange-correlation functional with basis set 6-311G** in a vacuum condition [16–18]. Accordingly, this approach was employed to evaluate the intrinsic character and the propensity of chemical reactivity, influencing their behaviour of binding, and thereby complementing the simulation studies such as molecular docking and dynamics. The electronic structure properties and the chemical reactivity of the compound (stomidazolone) were analyzed on the basis of frontier molecular orbital (FMO) theory. In the analysis, the spatial distributions of the frontier molecular orbitals derived from the highest occupied molecular orbital (HOMO) and the lowest unoccupied molecular orbital (LUMO) energies were shown to identify the regions related to LUMO (electron-accepting) and HOMO (electron-donating) in the optimized molecular geometry, while the energy band gaps (ΔEgap) between these LUMO and HOMO orbitals was used to evaluate the chemical reactivity and kinetic stability [19]. To further rationalize the chemical reactivity pattern, global reactivity descriptors were elucidated within the Koopmans’ theorem framework. These descriptors include ionization potential, electron affinity, chemical hardness, electronegativity, and electrophilicity index, estimating the tendency of a given molecule to lose or gain electrons, charge transfer resistance, electronic attraction, and molecular propensity to undergo electrophilic reactions. To ascertain the relative nucleophilic and electrophilic zones, Molecular Electrostatic Potential (MEP) has been mapped, which characterized the polarity of a molecule [20,21]. In the context of this study, these DFT-based calculations were performed to characterize the intrinsic electronic behavior of stomidazolone, providing a theoretical foundation for understanding how its charge distribution, orbital energy levels, and reactive sites contribute to its binding affinity and inhibitory interaction with MUTE [22–24].

### 2.2 Protein preparation

This study investigates the bHLH transcription factor MUTE, which contains a C-terminal ACTL domain. The three-dimensional structure of MUTE was obtained from the AlphaFold [25] Protein Structure Database (ID: AF-Q9M8K6) and used as the starting model for subsequent analyses. An internal validation of MUTE protein model predicted by Alphafold, showed a high confidence score (pLDDT>90) per-residue with low PAE (Predicted Alignment Error), reflecting a well-structured protein fold suitable for further studies. The average pLDDT values for the key residues within the binding region and ACTL domain were noted to be 72.26 with low PAE score, placing these residues within the “confident” prediction range. During the protein preparation process, several refinements were carried out, including the addition of missing residues and atoms, as well as adjustments to bond orders and formal charges. Hydrogen atoms were added using the Protonate3D algorithm [26]. For protonation, electrostatics were calculated using the Generalized Born Volume Integral (GB/VI) method, with a solvent dielectric constant set to 80. Cutoff distances for van der Waals and electrostatic interactions were defined as 10 Å and 15 Å, respectively [27]. Partial atomic charges [28] were assigned using the AMBER99 force field implemented in the 2019 version of the Molecular Operating Environment (MOE) software suite [29].

### 2.3 Molecular docking and identification of conserved residues

Molecular docking was performed to predict the preferred binding orientation and interaction profile of stomidazolone within the MUTE protein’s active site. The three-dimensional structure of the MUTE protein was imported into the docking interface of MOE for simulation and the binding site information was abstracted from the work of Nakagawa et al. Docking was carried out using the Triangle Matcher algorithm for ligand placement, combined with the London dG scoring function and an Induced Fit protocol to account for receptor flexibility. Stomidazolone conformers were generated using the MMFF94x force field [30], producing 50 distinct conformations while keeping all other settings at their default values. Prior to docking, the protein complex was prepared using the Protonate3D tool and refined with the AMBER99 force field available in MOE. This preparation included addition of hydrogen atoms, correction of bond orders, assignment of partial charges, and energy minimization until the RMSD was reduced to ≤ 0.3 Å. Among the generated ligand poses, the one with the lowest docking score, indicating the highest predicted binding affinity, was selected for detailed analysis of its binding pose and interaction with the MUTE protein. Docking was used to predict the orientation, and complementarity between stomidazolone and MUTE, providing a structural basis for understanding its binding mechanism. The resulting docked complex was subsequently used as the starting conformation for molecular dynamics simulations, allowing further validation of the interaction stability observed in the docking phase.

Additionally, Multiple sequence alignment (MSA) was used to compare and align common bHLH proteins involved in stomatal differentiation and regulation, including MUTE, FAMA, and SPCH to identify regions of similarity and evolutionary conserved residues, in Arabidopsis thaliana and other plant species including Arabidopsis lyrata, Brassica oleracea, Solanum lycopersicum, and Zea mays. MSA was carried out using Clustal X (version 2.1). MSA plays a crucial role in understanding protein structure-function relationships, and guiding drug discovery. Sophisticated scoring schemes and gap penalty strategies ensure biologically meaningful alignments.

### 2.4 All-atom molecular dynamic simulation protocol

All-Atom Molecular Dynamics Simulations [31] were carried out to assess the binding and stability of the stomidazolone with the MUTE protein while the Apo form of MUTE was used for comparison of the dynamics. MD simulations allow the system to evolve over time, capturing physiological motions and interactions, providing atomic-level insights into the stability, flexibility, and conformational dynamics of the protein-ligand complex that cannot be captured by static structural models. Stability metrics like RMSD, RMSF, RoG offer critical insights into the into the dynamic behavior of the stomidazolone–MUTE complex at the atomic level and how stomidazolone maintains stable interactions within the MUTE binding pocket.

The simulations were run on an NVIDIA RTX 3080 GPU using the CUDA-accelerated version of GROMACS [32]. Protein topologies were established using the AMBER99SB-ILDN force field, while ligand topologies were produced with ACPYPE. The catenated systems comprising of MUTE and stomidazolone, were solvated in a cubic box with the TIP3P water model and periodic boundary conditions [33–35]. To ensure system neutrality and mitigate undesirable edge effects, counterions were added in a 0.15 M NaCl solution, and periodic boundary conditions were applied. All systems underwent energy minimization using the steepest descent algorithm, with a convergence limit of 5000 kJ·mol ⁻ ¹·nm ⁻ ¹, to remove possible steric clashes. Following minimization, systems were equilibrated for 5000 ps in the NVT ensemble (isothermal-isochoric) at 300 K, followed by 2000 ps in the NPT ensemble (isothermal-isobaric) at 1 bar to stabilize pressure and ensure overall system stability. Production MD simulations were performed using the Leapfrog integration algorithm with a time step of 2 fs for accurate resolution of atomic motions. Pressure was controlled using the Parrinello-Rahman barostat, and temperature was controlled with the velocity-rescale thermostat [36]. A cutoff distance of 1.0 nm was applied for short-range van der Waals interactions, while long-range electrostatics were calculated using the Particle Mesh Ewald (PME) method. The MD setup used for this study is detailed in S1 Table. Trajectory analysis was performed using Visual Molecular Dynamics (VMD) [37]. Standard stability metrics, such as RMSF, RMSD, and radius of gyration (RoG), were calculated to assess both global and local dynamics of the systems. All graphs were generated using Xmgrace, and molecular interaction diagrams were created with Chimera software [38,39].

### 2.5 Mapping cross-correlation networks

Dynamic Cross-Correlation Matrix (DCCM) visualizes the temporal correlations in the movement of amino acid residues. This analysis was performed to compare the Cα atoms across all studied systems, allowing us to assess the continuous correlation of domain movements and to assess whether ligand binding affects the dynamic coupling between the ACT-Like (ACTL) and DNA-binding (bHLH) domains of MUTE. Under native conditions, these domains are expected to move in a correlated manner to enable efficient heterodimerization and transcriptional activity. Assessing changes in their correlated motion upon stomidazolone binding helps reveal whether the ligand dampens interdomain communication, offering insight into its potential inhibitory effect. Utilizing 300 ns trajectories, the DCCM analysis focused on the cross-correlation of the displacements of backbone Cα atoms. In this context, C_ij_ represents the correlation coefficient between atoms i and j, mathematically [40].


$$C_{ij}=\frac{\left\langle \Delta r_{i}.\Delta r_{j}\right\rangle }{\sqrt{\left\langle \Delta r_{i}.\Delta r_{i}\right\rangle \;\left\langle \Delta r_{j}.\Delta r_{j}\right\rangle }}$$

In the notation ⟨⟩, the time average is taken over the entire trajectory. Δr_i_ and Δr_j_ represent the displacement vectors, calculated as the difference between the instantaneous and average positions of the i^th^ and j^th^ atoms. The DCCM value ranges from −1 to +1, with C_ij_ > 0 indicating positive correlation (same direction) between the i^th^ residue and the j^th^ residue and C_ij_ < 0 indicating negative correlation (opposite direction). Bio3D R package was used for DCCM analysis and plotting.

### 2.6 Principal Component Analysis (PCA)

Principal component analysis was conducted to explore the dominant motion patterns and collective conformational changes within the stomidazolone–MUTE complex during the molecular dynamics simulations. By reducing the system’s dimensionality, PCA helps identify the essential dynamic modes that contribute most to the protein’s structural fluctuations, thereby revealing how ligand binding influences MUTE’s overall conformational stability and flexibility. Prior to analysis, trajectory alignment was performed as a standard preprocessing step. The Essential Dynamics (ED) method was then used to diagonalize the covariance matrix and obtain eigenvectors, eigenvalues, and their corresponding projections. PCA analysis was carried out using the MDAnalysis toolkit, focusing on the first two principal components. Additionally, these components were used to construct the system’s free energy landscape [41,42].

### 2.7 Free Energy Landscape (FEL)

Free energy landscape analysis was performed to visualize the energetic stability and conformational states of the stomidazolone–MUTE complex across the simulation trajectory. The FEL profile allows identification of low-energy basins corresponding to the most stable conformations, providing deeper insight into the thermodynamic favorability and binding stability of stomidazolone within the MUTE active site. The free energy landscape was generated using the gmx sham module in the GROMACS soft-ware suite, following the assessment of conformational space and molecular motions revealed by the simulations. The landscape represents potential protein conformations during molecular dynamics, based on Gibbs free energy and the first two principal components.


$$\Delta G=-K_{B}lnP\;\left({PC}_{\text{1}},{PC}_{\text{2}}\right)$$

In this context $P\left({PC}_{\text{1}},{PC}_{\text{2}}\right)$ denotes the probability distribution of conformations along the first two principal components, while $K_{B}$ and *T* refer to the Boltzmann constant and absolute temperature, respectively. This landscape visually maps the system’s energy distribution, providing insight into molecular kinetics and thermodynamic stability by highlighting various energy minima and their associated conformational states.

This analysis was conducted to characterize the conformational stability and energy distribution of MUTE in its Apo and ligand-bound states. By mapping free energy minima, we aimed to identify dominant stable conformations and assess whether stomidazolone binding stabilizes or restricts specific structural states relative to the Apo form. The FEL also serves as an energetic validation of docking and MD results, confirming the thermodynamic consistency of the observed stable binding poses and reduced flexibility upon ligand association.

## 3. Results and discussion

### 3.1 Geometry optimization

**Fig 1:**
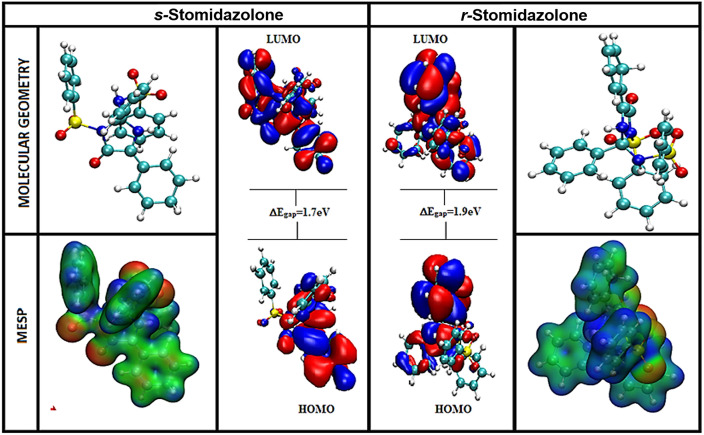
Visualization of Optimized molecular geometry (blue, red, cyan, yellow colours indicate nitrogen, oxygen, carbon and sulfur atoms), Molecular electrostatic potential (MESP), and Frontier molecular orbital (HOMO-LUMO) of r-stomidazolone and s-stomidazolone.

The isosurface plot of frontier molecular orbitals, HOMO (electrons donating) and LUMO (electron accepting) orbitals, where the electron distribution phases as positive (blue) and negative (red) lobes were visualized, within the r and s configuration of stomidazolone (Fig 1). The calculated ΔEgap (energy difference) of 1.72 eV and 1.9 eV for the s and r stomidazolone, respectively, indicates the tendency to undergo excitation of electrons from the orbitals (HOMO to LUMO) energy state, suggesting higher chemical reactivity on smaller band gaps and lower chemical reactivity on larger band gaps. Comparatively, a smaller energy gap of s stomidazolone (1.72eV) facilitates optimal transfer of charge, reflecting polarization effect and better chemical reactivity, thereby supporting an enhanced adaptability of a ligand within the protein (MUTE) binding site, presuming to be consistent with its active configuration as biologically observed in various plant systems.

### 3.2 Frontier molecular orbitals

The isosurface plot of frontier molecular orbitals represented the red and blue lobes as negative and positive transfer regions of charges in r and s-stomidazolone molecule (Fig 1). These spatial distributions of the charge transfer are essential to pinpoint the possible sites of nucleophilic and electrophilic concentration, often referred to as LUMO and HOMO. A band gap (ΔEgap) between the orbitals (LUMO-HOMO) energy levels determines the chemical reactivity potential of the molecular system, suggesting higher chemical reactivity on smaller band gaps and lower chemical reactivity on larger band gaps. The under-study molecular systems of s-stomidazolone have shown a smaller energy gap of 1.72eV as compared to r-stomidazolone energy gap of 1.9eV, suggesting that s-stomidazolone is more chemically reactive than r-stomidazolone.

### 3.3 Molecular Electrostatic Potential (MESP) map

On a molecular electrostatic potential (MESP) map, the electrophilic and nucleophilic zones have been graded by colours. The red colour indicates the negative potential (nucleophilic zone) while the blue and green colours represent the positive (electrophilic zone) and neutral potentials, respectively, as shown in Fig 1. The MESP map around the heteroatoms has shown different polarization, a high probability of negative potential observed over the oxygen atoms attached to nitrogen atoms of sulfonyl groups and the carbonyl oxygen, corresponding to strong nucleophilic character, and were predicted to show interaction with the positively charged amino-acid residues either through electrostatic or hydrogen bond interaction. While the electrophilic zone is localized on the sulfur center and around the imidazolone nitrogen, notably, a positive potential on the sulfur center reflects the deficiency of electrons, likely due to adjacent site electronegative oxygen atom substituents. Over the aromatic carbon skeleton the neutral zone (green colour) was observed, and packs against the amino acid residues that are hydrophobic in nature, implying the stabilization of van der Waals. Collectively, the MESP map suggests favourable interactions with the crucial amino acid residues of the MUTE protein.

### 3.4 Quantum chemical descriptors

The global reactivity descriptors in terms of chemical hardness(η), Electrophilicity index (ω), chemical potential(μ), ionization potential (IP), and electron affinity (EA) enlists in S2 Table, are all correlated with the frontier molecular orbitals (LUMO-HOMO) energies. According to Koopmans’ theorem, the negative of the calculated HOMO and LUMO energies have been used to estimate the ionization potential and electron affinity. Based on the resulting data, comparatively, for the s-stomidazolone, the lower value of chemical hardness (η) reflects the lower resistance in transfer of charge, facilitating the higher chemical reactivity, suggesting that compound (s-stomidazolone) can readily undergo the delocalization of electrons, and may participate in molecular interaction. The lower value of chemical potential (μ) estimates the higher nucleophilic tendency; correspondingly, the higher value of electronegativity (χ) indicates the electron-withdrawing character. Moreover, the electrophilicity index (ω) quantifies the stability of a molecular system (r and s-stomidazolone) upon accepting an electron; that is, the process is energetically favourable when the (ω) value is higher, reflecting better stability, while a lower value indicates no significant stability and less reactivity. A relatively lower electrophilicity index was calculated for s-stomidazolone, signifying its better stability as an electron acceptor. Collectively, the nucleophilic (chemical potential) and electrophilic (electrophilicity index) potential of s-stomidazolone reflect effective participation during the biological interaction.

### 3.5 Interaction profile of stomidazolone

**Fig 2:**
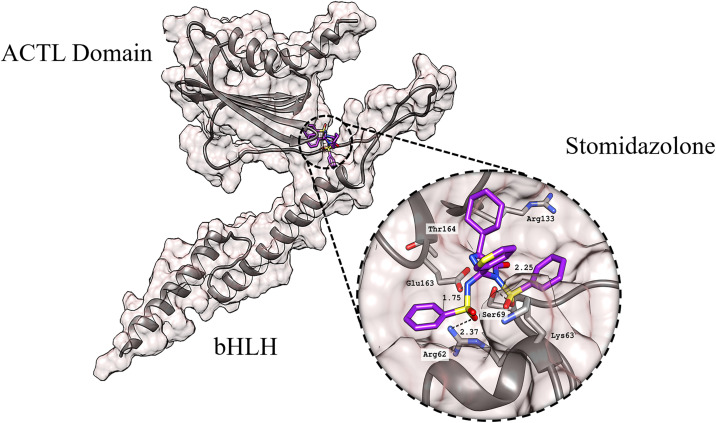
The binding of stomidazolone (purple) with the MUTE protein. The protein structure is depicted using a combination of surface and ribbon representations, with a magnified view of the binding site for enhanced clarity. The ACTL and bHLH domains are labeled. The dotted black lines represent hydrogen bonding. Image was rendered utilizing UCSF Chimera.

Molecular docking was employed to investigate the binding of stomidazolone with the MUTE protein to provide insight into their comprehensive binding interaction. In this context, structural coordinates of the MUTE protein were used as the foundational framework. Stomidazolone exhibited a high binding affinity for MUTE owing to a network of critical interactions, as illustrated in Fig 2. The interactions of stomidazolone with MUTE are stabilized by several key residues. Hydrogen bonds, represented by black dotted lines, play a crucial role in ligand anchoring: Arg62 contributes a hydrogen bond at 2.37 Å, Ser69 forms hydrogen bonds at 2.25 Å, and Glu163 forms an additional hydrogen bond at 1.75 Å. Other residues such as Thr164, Arg133, and Lys63 are positioned near the ligand, contributing additional stabilization through short-range van der Waals and electrostatic interactions, as reflected in the favorable docking energy profile.

Biologically, these residues are located within or adjacent to the ACT-Like (ACTL) domain, which plays a regulatory role in MUTE’s structural conformation and potential dimerization interface with partner proteins such as SCRM/SPCH/FAMA. The engagement of stomidazolone at Arg62 and Ser69 residues implicated in maintaining local folding and intra-domain stability, suggests a mechanism by which ligand binding could alter domain flexibility or disrupt protein-protein interactions essential for stomatal lineage progression. Similarly, Glu163 and Thr164, situated near the C-terminal region of the ACTL domain, are conserved residues (S1 Fig) that may participate in stabilizing the transcriptionally active conformation of MUTE; thus, their engagement by stomidazolone could contribute to inhibition of functional dimer formation or DNA binding. Notably, these interactions overlap with the binding determinants reported by Nakagawa et al.

This structural depiction underscores the molecular recognition of stomidazolone by the protein, emphasizing a strong hydrogen bonding network that contributes to ligand stability. The multiple interactions observed suggest a mechanistic basis for modulation of MUTE activity, potentially influencing stomatal differentiation signaling pathways. Corresponding to, the multiple sequence alignment of the bHLH proteins involved in stomatal differentiation and regulation, including MUTE, FAMA, and SPCH proteins showed significant conservation of the functionally important ACTL domain, notably, the amino-acid residues within the binding site Arg62, Lys63, and Thr164, reflecting a crucial role in the maintenance of structural integrity and functional activities in Arabidopsis thaliana with bHLH protein orthologs found in other plant species such as Arabidopsis lyrata, Brassica oleracea, Solanum lycopersicum, and Zea mays (S1 Fig), reinforcing the biological relevance of these interaction hotspots for potential inhibitor design.

### 3.6 Assessment of structural integrity

Root Mean Square Deviation (RMSD) is a widely used metric in molecular dynamics simulations to assess the extent of structural deviation between the initial (native) and final conformations of a protein. It offers important insights into the structural stability and dynamic behavior of biomolecular systems. In general, lower RMSD values reflect higher structural stability, while elevated values may indicate increased flexibility or instability. To evaluate the structural integrity and overall dynamics of both the native and Stomidazolone-bound systems, RMSD values were calculated over the course of 300 ns simulation trajectories for each complex.

**Fig 3:**
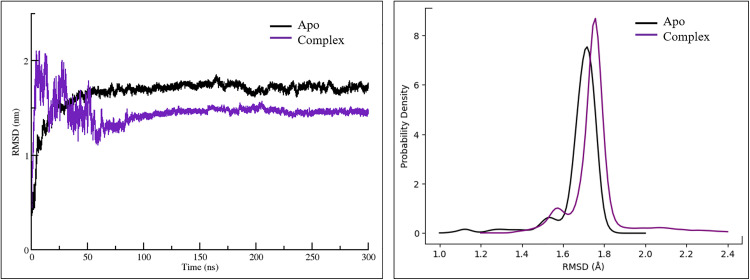
A) RMSD plots of the 300 ns simulated trajectories of Apo (unbound) and complex (ligand-bound) state of the MUTE protein. **B)** Probability density plot based on the RMSD distribution of the protein backbone.

As shown in Fig 3A, in the initial phase (0–50 ns), both the Apo and complex states exhibit rapid fluctuations as the protein undergoes structural adjustments to stabilize in the simulation environment. The Apo form (black) shows higher fluctuations, with an RMSD exceeding 2 nm at the start before stabilizing around 1.75–2.0 nm. In contrast, the complex form (purple) initially fluctuates more than the Apo state but stabilizes at a lower RMSD (~1.25 nm) after 100 ns, suggesting that ligand binding enhances structural rigidity. Over the remaining simulation period (100–300 ns), the Apo state maintains higher RMSD values and greater fluctuations, indicating a more flexible and dynamic structure. The complex state stabilizes earlier and remains more compact, signifying that ligand binding reduces conformational flexibility and promotes a more stable structural conformation. The average RMSD values for the Apo and complex state were noted as 1.65 ± 0.18 and 1.45 ± 0.12 nm respectively.

This probability density graph as shown in Fig 3B, represents the Root Mean Square Deviation (RMSD) distribution for the Apo and the complex state (bound to stomidazolone). Both states exhibit RMSD peaks around 1.7–1.8 nm, suggesting that both structures are relatively stable and do not undergo significant conformational changes. However, the peak for the complex state is slightly shifted and sharper compared to the Apo state, indicating that the protein in the bound state (with stomidazolone) adopts a more restricted and stable conformation. In contrast, the Apo state displays a broader distribution, consistent with greater conformational diversity.

From a biological perspective, the observed reduction in flexibility upon stomidazolone binding implies that the ligand stabilizes MUTE in a conformationally constrained or inactive state. Since MUTE’s functional activity depends on conformational adaptability for heterodimerization with SCRM and subsequent transcriptional activation, the enhanced rigidity in the complex may hinder these dynamic interactions, thereby supporting a potential inhibitory mechanism of stomidazolone.

### 3.7 Evaluation of structural flexibility

**Fig 4:**
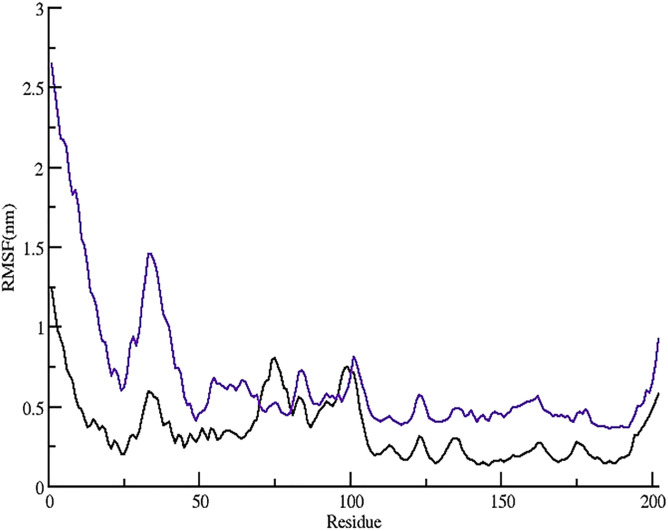
RMSF plot of the 300 ns simulated trajectories of the Apo (unbound) and complex (ligand-bound) state of the MUTE protein.

To assess the intrinsic flexibility of both the apo and ligand-bound systems throughout the 300 ns molecular dynamics simulation, root mean square fluctuation (rmsf) analysis was performed. Rmsf provides residue-level insights into atomic mobility, where higher values correspond to greater flexibility and potentially reduced stability. The rmsf plot depicts how individual residues deviate from their average positions over time, with values reported in nanometers. By comparing the rmsf profiles of the unbound (black) and stomidazolone-bound (purple) forms of the protein, the influence of ligand binding on protein dynamics can be clearly observed, as illustrated in Fig 4.

Residues 62, 63, 69, 133, 163, and 164 exhibit notable differences in flexibility between the two states. In the complex state, residues 62 and 63 show slightly elevated RMSF values compared to the Apo state, suggesting that ligand binding enhances their mobility. Residue 69 also displays increased fluctuations in the complex form, indicating that this region becomes more flexible upon ligand interaction. Conversely, residue 133 shows minimal differences between the Apo and complex states, indicating that its flexibility remains largely unaffected by binding. Residues 163 and 164, however, demonstrate a marked increase in fluctuation in the complex form, signifying that ligand binding enhances their dynamics. This increased flexibility could be associated with structural adjustments necessary for effective ligand accommodation or allosteric regulation. Overall, the graph suggests that while ligand binding stabilizes the overall protein structure, it selectively increases the flexibility of specific residues, potentially influencing the protein’s functional dynamics and interaction capabilities. The average RMSF values for the Apo and the complex states were observed as 0.35 ± 0.19 and 0.67 ± 0.41 nm respectively.

### 3.8 Assessment of structural compactness

To further validate the fluctuation patterns and structural changes observed in the simulation, a gyration analysis was conducted, an essential approach for evaluating a protein’s conformational flexibility in solution. The Radius of Gyration (Rg) serves as an important indicator of structural compactness, defined as the mass-weighted root mean square distance of a protein’s atoms from its center of mass. Consistent Rg values suggest a well-folded and stable structure, whereas increased Rg values under dynamic conditions may reflect partial unfolding and reduced stability. To gain deeper insights into conformational behavior, we examined the time-dependent profile of Rg across the simulation. This analysis provided information on folding dynamics and the impact of ligand binding on the receptor’s structural compactness. Stable Rg trajectories are indicative of a correctly folded protein, whereas continuous fluctuations imply structural instability, highlighting Rg as a reliable marker for assessing protein integrity over time.

**Fig 5:**
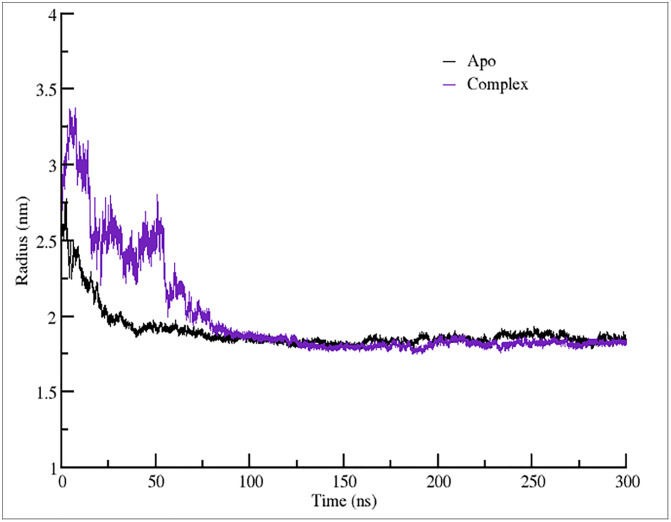
Rg plots of the 300 ns simulated trajectories of the Apo (unbound) and complex (ligand-bound) state of the MUTE protein.

The Rg vs time plot as shown in Fig 5, representing the Apo (unbound) state, shown in black, and the complex (bound) state, shown in purple. Initially, the complex form exhibits a higher Rg (~3.5 nm) compared to the Apo form (~3.0 nm), indicating that the bound state starts in a more relaxed conformation with both trajectories showing a rapid decrease in Rg within the first 50 ns, suggesting an initial structural relaxation phase. After approximately 100 ns, the Rg stabilizes around ~2.0 nm for both states, with no significant fluctuations thereafter, indicating that the system has reached equilibrium. The final Rg values for the complex state appear slightly lower than the Apo state, implying that binding does not significantly alter the overall compactness of the structure in the long run. This indicates that while ligand binding initially perturbs the protein’s structure, the equilibrium compactness reflects a stabilized state rather than unfolding or collapse. The absence of large differences suggests that MUTE retains its overall fold, but the slightly altered Rg profile may correspond to restricted conformational sampling and reduced flexibility upon binding, consistent with the FEL and PCA results showing narrower conformational space exploration. Functionally, this implies that stomidazolone binding stabilizes MUTE in a more rigid state, potentially limiting conformational transitions necessary for activation. The average Rg values for Apo and the complex system were observed as 1.89 ± 0.13 and 1.97 ± 0.33 nm respectively. A statistical analysis of RMSD and Rg values observed is vailable in S3 Table.

### 3.9 Exploration of solvent accessible surface area

**Fig 6:**
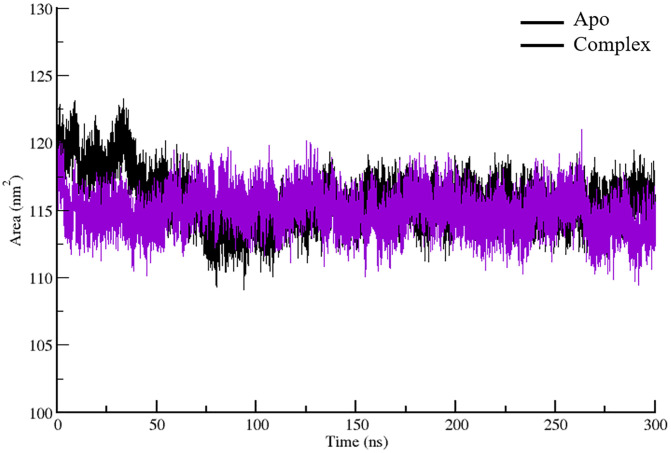
SASA plot of the 300 ns simulated trajectories of the Apo (unbound) and complex (ligand-bound) state of the MUTE protein.

Solvent Accessible Surface Area (SASA) is a measure of the surface area of a molecule that is exposed to the surrounding solvent. It quantifies the extent to which a biomolecule, such as a protein, interacts with water or other solvent molecules. SASA is an important parameter in molecular dynamics simulations, as it provides insights into protein folding, stability, and binding interactions. A higher SASA indicates a more exposed and extended structure, while a lower SASA suggests a more compact and buried conformation, often due to ligand binding or structural rearrangements. The Solvent Accessible Surface Area (SASA) vs. Time (ns) plot represents the Apo (unbound) state, shown in black, and the complex (bound) state, shown in purple (Fig 6). Initially, the Apo state exhibits a slightly higher SASA (~120 nm²) compared to the complex state (~115 nm²), indicating that the unbound protein is more exposed to the solvent. Over time, both systems fluctuate but remain relatively stable, with the complex consistently maintaining a slightly lower SASA than the Apo state. This suggests that binding reduces the overall solvent exposure of the protein. The complex initially had a larger radius but quickly contracted to a stable, more compact conformation. This structural compactness naturally results in a reduced SASA, as a tightly packed structure has fewer exposed residues available for solvent interaction. In contrast, the Apo form maintains a slightly more expanded structure, leading to greater solvent accessibility. Overall, the combination of the Rg and SASA data suggests that ligand binding induces a more compact and less solvent-exposed conformation. Notably, this reduction in solvent exposure likely involves residues within or adjacent to the ACTL and DNA-binding domains, which are key for dimerization and transcriptional regulation. Decreased SASA in these regions may therefore restrict access to interaction partners or the DNA-binding interface, potentially modulating MUTE’s ability to form active complexes during stomatal lineage progression. This could imply that the binding event enhances structural stability and reduces solvent accessibility, potentially influencing the protein’s functional dynamics and interaction with other molecules.

### 3.10 Betweenness Centrality (BC) analysis

The betweenness centrality (BC) plot illustrates the significance of individual residues in facilitating communication within the protein structure. BC values represent the number of shortest paths that pass through each residue, highlighting key positions that contribute to inter-domain signaling and structural stability.


$$BCv=\frac{\text{1}}{m}\sum \begin{array}{c} m\\ i=\text{1} \end{array}\sum \begin{array}{c} s,t\epsilon v \end{array}\frac{\sigma i(s,t\vee v)}{\sigma i(s,t)}$$

**Fig 7:**
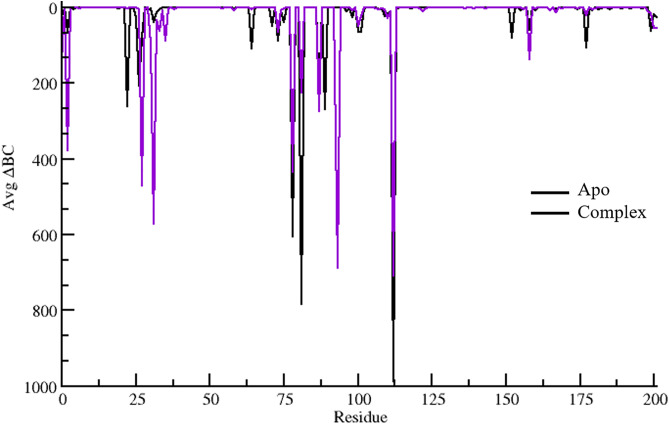
Betweeness centrality plot of the 300 ns simulated trajectories of the Apo (unbound) and complex (ligand-bound) state of the MUTE protein.

Betweenness centrality quantifies the proportion of all shortest paths between node pairs that pass through a specific node v. Here, σi(s,t|v) represents the number of shortest paths that include residue v, while σi(s,t) denotes the total number of shortest paths in the graph, both measured at a given time i. This value is then averaged across all m frames for each residue. The plot (Fig 7) compares the Apo (black) and complex (purple) states, providing insights into how ligand binding affects the communication network of the protein.

In both states, several residues exhibit sharp decreases in BC, indicating their pivotal roles in mediating connectivity. Notably, residues around positions 62, 63, 69, 133, 163, and 164 show distinct patterns. Residues 62 and 63 display a slight reduction in BC upon ligand binding, suggesting a shift in their communicative importance. Residue 69 exhibits a more pronounced change, potentially reflecting an altered pathway in the complex state. Similarly, residue 133 experiences a change in BC, albeit to a lesser extent, implying a moderate influence on inter-domain interactions. The most significant differences are observed in residues 163 and 164, where BC values show a marked deviation between the Apo and complex forms. This suggests that these residues play a crucial role in communication, with ligand binding either enhancing or redistributing their connectivity within the network. The overall trend indicates that while the global communication network remains largely conserved, certain residues undergo notable shifts in importance, which could be key to understanding allosteric regulation and functional modulation upon ligand binding.

### 3.11 Hydrogen bond interaction analysis

**Fig 8:**
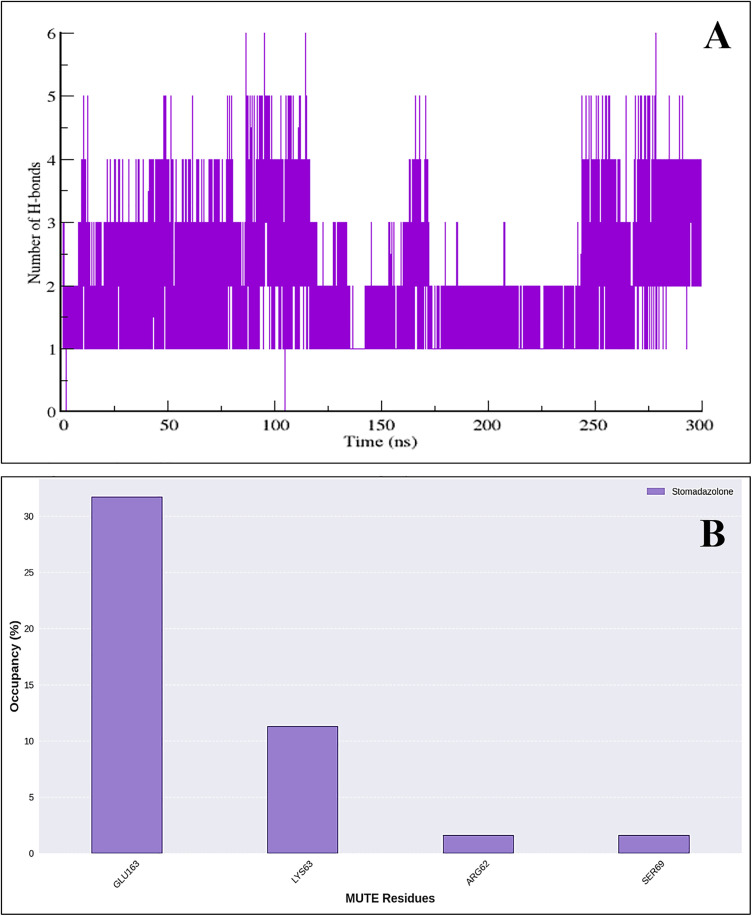
A) Number of hydrogen bonds of the MUTE protein as the fraction of conformations over 300 ns. **B)** Hydrogen bond occupancy plot with respect to residues of the MUTE protein.

The plot representing the number of hydrogen bonds formed and retained over the period of 300 ns between stomidazolone and the MUTE protein is shown in Fig 8A. Throughout the simulation, the number of hydrogen bonds fluctuates between 0 and 5 with a mean bond number of 1.93 + /- 0.96, indicating dynamic interactions between stomidazolone and the MUTE protein. The plot reveals distinct periods of stable hydrogen bonding, particularly in the initial 100 ns and the final 50 ns, where bond occupancy is relatively high, with consistent formations of around 3–4 hydrogen bonds. The plot suggests that stomidazolone maintains a moderate-to-strong binding affinity with the MUTE protein, characterized by varying hydrogen bond occupancy. For further analysis, the bar plot (Fig 8B) represents the residue specific hydrogen bond occupancy of key MUTE protein residues in interaction with stomidazolone, providing insights into residue-specific interactions that are crucial for understanding the binding and stability of ligand-protein interaction. The occupancy percentage reflects how frequently each residue engages in hydrogen bonding with the ligand during the simulation while the x-axis lists the MUTE residues involved in these interactions. Among these residues, Glu163 exhibits the highest hydrogen bond occupancy, surpassing 30%, suggesting a strong and consistent interaction with stomidazolone. Lys63 follows with an occupancy of around 10%, indicating a moderate yet significant contribution to ligand binding. In contrast, Arg62 and Ser69 display much lower occupancies, each contributing only a small fraction of the total hydrogen bonding interactions. This distribution highlights the dominant role of Glu163 in stabilizing stomidazolone within the binding pocket, with Lys63 playing a secondary but notable role. The relatively low occupancies of Arg62 and Ser69 suggest transient or weaker hydrogen bonding interactions.

### 3.12 Analysis of dynamic cross-correlation networks

**Fig 9:**
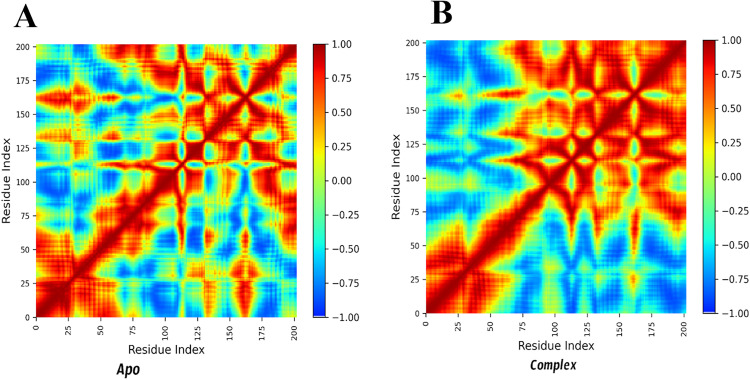
The Dynamic Cross-Correlation Matrix (DCCM) plots of the A) Apo (unbound) and B) complex (ligand-bound) states. The color scale represents correlation coefficients, where red (values close to +1) indicates strong positive correlations, blue (values close to −1) signifies strong negative correlations, and yellow/green (values near 0) suggests minimal or no correlation between residue movements.

The Dynamic Cross-Correlation Matrix (DCCM) plots (Fig 9) presented here illustrate the correlated and anti-correlated motions between different residues of the protein in both the Apo (unbound) and complex (ligand-bound) states. The color scale represents correlation coefficients, where red (values close to +1) indicates strong positive correlations (residues moving in the same direction), blue (values close to −1) signifies strong negative correlations (residues moving in opposite directions), and yellow/green (values near 0) suggests minimal or no correlation between residue movements.

In the Apo state (Fig 9A), the correlation pattern exhibits more dispersed and varied interaction regions, suggesting greater flexibility and dynamic movement among the residues. The absence of a bound ligand allows the protein to sample a wider conformational space, leading to more heterogeneous correlations. The complex state (Fig 9B), on the other hand, displays a more structured and pronounced correlation pattern, particularly along the main diagonal, indicating that ligand binding stabilizes the protein structure and promotes more coordinated motions between residues. The presence of more intense red regions suggests stronger intramolecular interactions, potentially leading to a more compact and functionally optimized conformation.

Specifically, residues 62 and 63 show an increase in positive correlations upon ligand binding, suggesting that they contribute to stabilizing intra-domain interactions within the complex. Residue 69, which exhibits weak correlations in the Apo state, shifts to stronger intra-domain positive correlations in the complex state, implying that ligand binding enhances coordinated movement in this region. Residue 133 shows increased cross-correlations with distant residues in the complex state, hinting at potential allosteric effects induced by ligand engagement. Similarly, residues 163 and 164 display stronger correlations with regions in the 100–150 range in the complex, reinforcing the idea that stomidazolone binding enhances cooperative motions and increases structural rigidity. These enhanced correlations between residues 62–69 (within the ACT-like domain) and 163–164 (near the putative DNA-binding interface) suggest the presence of an allosteric communication pathway that is disrupted or dampened upon ligand binding. The correlation changes align with Radius of Gyration (Rg) and Solvent-Accessible Surface Area (SASA) results, both indicating a shift toward a more ordered conformation. The decreased Betweenness Centrality (BC) values for residues 62, 63, 69, 133, 163, and 164 further support a decline in long-range communication across the protein, consistent with disruption of allosteric signaling. Notably, Glu163, which exhibits high occupancy and strong correlated motion, is conserved among MUTE orthologs, highlighting its potential as a critical anchoring site for inhibitor design and as a determinant of structural coupling following stomidazolone interaction.

### 3.13 Conformational motions and thermodynamic landscape

**Fig 10:**
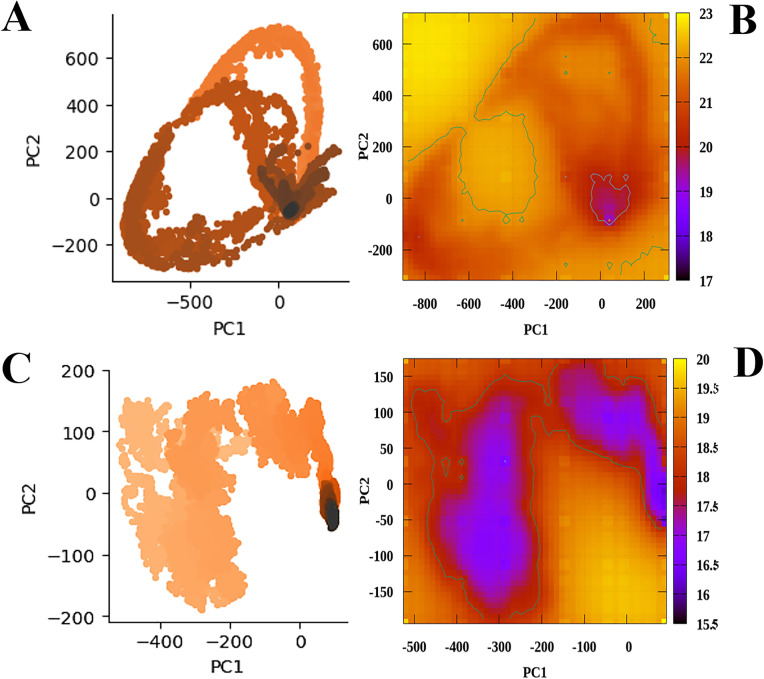
A side by side representation of the PCA and Free Energy Landscape (FEL) profiles of the Apo (unbound) (A, B) and complex (ligand-bound) state of the MUTE protein (C, D).

A side by side representation of the PCA and Free Energy Landscape (FEL) profiles for both protein systems are illustrated in Fig 10(A–D), highlighting their conformational and thermodynamic stability. The principal component analysis (PCA) plot provides a detailed visualization of the significant conformational variations within the native system across the first two principal components (PC1 and PC2). The clustering of points in this plot highlights areas of high configurational sampling, with dense, darker regions representing conformations that the system occupies more frequently. In contrast, the lighter areas show less frequently sampled states, indicating conformational transitions. The free energy landscape plot on the right provides insights into the stability and energy distribution of these conformations. The color gradient represents the free energy values, with lower energy regions depicted in purple and higher energy areas in yellow.

As shown in (Fig 10A), the distribution of points suggests a diverse range of sampled conformations, with a denser cluster forming around the region close to the origin, particularly between approximately −200–0. This clustering may indicate a preferred conformational state, while the broader spread signifies transitions between different structural states. The free energy landscape plot (Fig 10B) shows a significant low-energy basin is observed around the range of approximately −200–0, aligning with the dense cluster in the PCA plot. This suggests that the Apo protein has a stable conformational state in this region, while the surrounding areas correspond to higher-energy, less stable conformations. The contour lines highlight the energy variations, reinforcing the presence of dominant basins that define the protein’s most favorable states.

The distribution of points for the complex system (Fig 10C) shows a more compact sampling of conformations compared to the Apo protein, indicating restricted motion upon complex formation. The densest cluster is located around the region close to 0 with most conformations ranging between −50 and 100. This clustering suggests that the complex protein predominantly adopts a limited range of conformations with fewer large-scale transitions. Notably, two distinct low-energy basins are observed (Fig 10D) between −400 and −100 and extending up to 150, indicating preferred and stable conformational states. These distinct minima may represent inactive or partially restricted conformations stabilized by stomidazolone binding, consistent with a mechanism of conformational inhibition. The contour lines outline the energy variations, reinforcing the presence of dominant energy minima that define the most favorable structural states for the complex protein.

## 4. Conclusion

Stomatal regulation plays a pivotal role in balancing plant growth and water conservation through a tightly orchestrated developmental program mediated by key bHLH transcription factors such as SPCH, MUTE, and FAMA. Previous studies identify stomidazolone, a doubly sulfonylated imidazolone compound, as a potent inhibitor of stomatal differentiation underscore the potential of small molecules to modulate these regulatory networks. Through a comprehensive in silico approach combining molecular docking, density functional theory (DFT), and all-atom molecular dynamics simulations, this study elucidates the molecular mechanism by which stomidazolone binds to the ACTL domain of MUTE, forming a stable complex as evident by the stability metrics such as RMSD, and the persistent hydrogen bonding involving Arg62 and Ser69, along with an additional stabilizing contribution from Glu163, collectively supporting the strong binding affinity of stomidazolone within the MUTE active site. By stabilizing MUTE in a restricted conformational ensemble through these interactions stomidazolone likely prevents its heterodimerization with SCRM, thereby blocking stomatal lineage progression. The computational insights obtained in this study complement existing in vivo and in vitro findings on stomatal differentiation. The observed stability of the MUTE–stomidazolone complex and the identified key interaction residues align with experimental evidence that disruption of MUTE–SCRM dimerization impedes stomatal lineage progression. While these computational results provide valuable molecular-level insights, they also highlight key directions for future work. Experimental validation, including cryo-EM or NMR structural validation, in vitro binding assays, site-directed mutagenesis of key MUTE residues involved in stomidazolone binding, and phenotypic analyses in transgenic lines, will be essential to confirm the proposed inhibitory mechanism. Furthermore, rational modification of the stomidazolone scaffold, guided by its binding interactions, could yield derivatives with improved specificity, bioavailability, or reduced phytotoxicity. Extending this approach to related bHLH transcription factors may uncover broader regulatory control points for the chemical modulation of stomatal development. This study underscores the conceptual novelty of employing small-molecule–based modulation of lineage-specific transcription factors as a strategy to influence plant developmental pathways. Such an approach provides a foundation for the synthetic control of plant developmental plasticity, potentially enabling the fine-tuning of physiological processes such as stomatal differentiation. While the implications for improving water-use efficiency and stress adaptation are promising, these outcomes remain hypothetical and require further experimental validation to establish their functional relevance. Moreover, practical translation of these findings will depend on addressing challenges such as ensuring target specificity to minimize off-target inhibition of other bHLH proteins, and optimizing compound stability, bioavailability, and delivery efficiency in planta. Recognizing these limitations emphasizes the need for continued experimental and formulation research to advance such chemical modulators toward field-applicable, sustainable agrochemical solutions. Importantly, this study provides the first mechanistic, residue-level insight into how stomidazolone engages the ACTL domain of MUTE, revealing the specific molecular interactions and dynamic features that underpin its inhibitory effect. These findings establish a foundational framework for understanding the mode of action of stomidazolone at atomic resolution, setting the stage for future experimental and rational design efforts directed towards the design of next-generation agrochemicals targeting transcriptional regulators of stomatal development, paving the way for selective, sustainable strategies to enhance crop performance and resilience under changing environmental conditions.

## Supporting information

S1 Fig Schematic representation of multiple sequence alignment (MSA) of MUTE ACTL domain. (DOCX)

S1 Table Details of MD Simulation Setup. (DOCX)

S2 Table Quantum chemical properties of Stomidazolone molecules. (DOCX)

S3 Table Statistical analysis of RMSD and RoG. (DOCX)
