# Supplementary material for: Computational elucidation of stomidazolone mediated inhibition of stomatal differentiation and its implication in plant developmental regulation
Source: PLoS One. 2026 Feb 10;21(2):e0329401. doi: 10.1371/journal.pone.0329401 (PMC12890161; doi:10.1371/journal.pone.0329401)
Supplement: S1 Fig — (DOCX) [file pone.0329401.s001.docx]

**
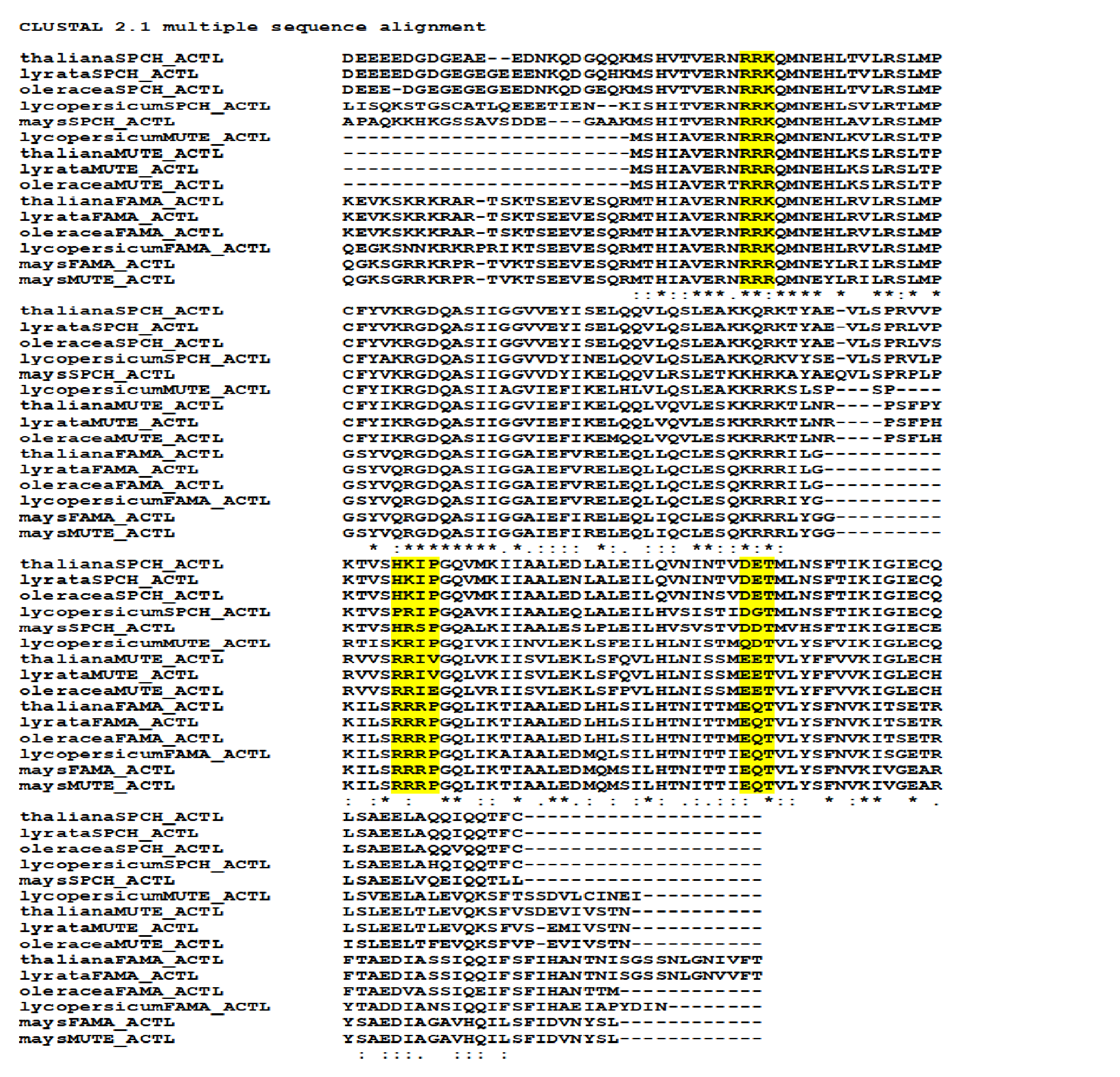
**

**Figure S1:** Schematic representation of Multiple sequence alignment (MSA) of MUTE ACTL domain.

The multiple sequence alignment of the bHLH proteins involved in stomatal differentiation and regulation, includes MUTE, FAMA, and SPCH proteins have shown significant conservation of the functionally important bHLH/ACTL domain in Arabidopsis thaliana and their bHLH protein orthologs, while other regions exhibit sequence variations, suggesting the evolutionary divergence. In addition, amino-acid residues of protein binding sites, highlighted in yellow colour, have shown significant conservation.
