# Supplementary material for: Computational elucidation of stomidazolone mediated inhibition of stomatal differentiation and its implication in plant developmental regulation
Source: PLoS One. 2026 Feb 10;21(2):e0329401. doi: 10.1371/journal.pone.0329401 (PMC12890161; doi:10.1371/journal.pone.0329401)
Supplement: S1 Table — (DOCX) [file pone.0329401.s002.docx]

**Table S1: Details of MD Simulation Setup.**

| Parameter | Value / Method | Justification / Reference |
| --- | --- | --- |
| Force field | AMBER99SB-ILDN | Accurate backbone and side-chain representation; suitable for protein domains.  Reference: Lindorff-Larsen, K., Piana, S., Palmo, K., Maragakis, P., Klepeis, J. L., Dror, R. O., Shaw, D. E. (2010). Improved side-chain torsion potentials for the Amber ff99SB protein force field. Proteins, 78(8), 1950–1958. DOI: 10.1002/prot.22711 |
| Energy minimization | Steepest Descents, Fmax < 1000 kJ·mol⁻¹·nm⁻¹ (2051 steps) | In order to remove steric clashes and structural relaxation. |
| Electrostatics | PME (Particle Mesh Ewald) | It captures the long range columbic interactions Reference: Darden, T., York, D., Pedersen, L. (1993). Particle mesh Ewald: An N·log(N) method for Ewald sums in large systems. J. Chem. Phys., 98(12), 10089–10092. DOI: 10.1063/1.464397 |
| Nonbonded cutoff | 10–12 Å | Standard in biomolecular simulations for accuracy and efficiency |
| Temperature coupling | 300 K (NVT/NPT ensemble(10ns each)) | Maintains physiological temperature; confirmed stable (300 ± 0.1 K) |
| Pressure coupling | 1 bar (Parrinello–Rahman) | Ensures correct solvent density and isotropic box scaling  Reference: Parrinello, M., Rahman, A. (1981). Polymorphic transitions in single crystals: A new molecular dynamics method. J. Appl. Phys., 52(12), 7182–7190. DOI: 10.1063/1.328693 |
| Integration step | 2 fs (LINCS constraints on H-bonds) | Stable integration; standard for all-atom MD  Reference: Hess, B., Bekker, H., Berendsen, H. J. C., Fraaije, J. G. E. M. (1997). LINCS: A linear constraint solver for molecular simulations. J. Comput. Chem., 18(12), 1463–1472. DOI: 10.1002/(SICI)1096-987X(199709)18:12<1463::AID-JCC4>3.0.CO;2-H |
