# Supplementary material for: Computational elucidation of stomidazolone mediated inhibition of stomatal differentiation and its implication in plant developmental regulation
Source: PLoS One. 2026 Feb 10;21(2):e0329401. doi: 10.1371/journal.pone.0329401 (PMC12890161; doi:10.1371/journal.pone.0329401)
Supplement: S2 Table — (DOCX) [file pone.0329401.s003.docx]

**Table S2**: Quantum chemical properties of Stomidazolone molecules.

| **Quantum Chemical Descriptors** | **Formula** | **s-Stomidazolone (eV)** | ***r-*Stomidazolone (eV)** |
| --- | --- | --- | --- |
| Energy gap (ΔE_gap_) | ΔE_gap_ = LUMO-HOMO | 1.728 | 1.923 |
| Electron affinity (EA) | EA = - (LUMO) | 0.008 | 0.013 |
| Ionization potential energy (IP) | IP= - (HOMO) | 0.072 | 0.084 |
| Chemical hardness (η) | $\left( n \right)=\left( n\frac{I-A}{2} \right)$ | 0.031 | 0.035 |
| Electronic chemical potential (μ) | $\left( n \right)=\left( n\frac{I-A}{2} \right)$ | -0.040 | -0.048 |
| Electrophilicity index (ω) | $\left( \omega\right)=\left( \omega\frac{\mu^{2}}{2\eta} \right)$ | 0.026 | 0.033 |
