# Supplementary material for: Computational elucidation of stomidazolone mediated inhibition of stomatal differentiation and its implication in plant developmental regulation
Source: PLoS One. 2026 Feb 10;21(2):e0329401. doi: 10.1371/journal.pone.0329401 (PMC12890161; doi:10.1371/journal.pone.0329401)
Supplement: S3 Table — (DOCX) [file pone.0329401.s004.docx]

**Table S3: Statistical Analysis of RMSD and Rg**

Structural dynamics were compared between the apo and complex forms using molecular dynamics–derived parameters. The root-mean-square deviation (RMSD) showed a significant increase upon complex formation (M_diff = 0.70 ± 0.27, Z = −27.39, p < 0.001, r = 0.87), indicating greater conformational deviation from the apo state. Similarly, the radius of gyration (Rg) was significantly higher in the complex (M_diff = 0.23 ± 0.12), t(99) = 19.11, p < 0.001, Cohen’s d = 1.91, suggesting an expansion in overall structural compactness. Together, these results demonstrate that ligand binding induces notable structural flexibility and expansion in the protein.

**For RMSD**

| **Tests of Normality** | | | | | | | |
| --- | --- | --- | --- | --- | --- | --- | --- |
|  | Kolmogorov-Smirnov^a^ | | | | Shapiro-Wilk | | |
|  | Statistic | | df | Sig. | Statistic | df | Sig. |
| RMSD_diff | .073 | | 1000 | .000 | .975 | 1000 | .000 |
| a. Lilliefors Significance Correction | | | | | | | |
| **Test Statistics^a^** | | | | | | | |
|  | | RMSD(Complex) - RMSD(Apo) | | | | | |
| Z | | -27.393^b^ | | | | | |
| Asymp. Sig. (2-tailed) | | .000 | | | | | |
| a. Wilcoxon Signed Ranks Test | | | | | | | |
| b. Based on negative ranks. | | | | | | | |

**For RoG**

| **Tests of Normality** | | | | | | | | | | | | | | | |
| --- | --- | --- | --- | --- | --- | --- | --- | --- | --- | --- | --- | --- | --- | --- | --- |
|  | | Kolmogorov-Smirnov^a^ | | | | | | | Shapiro-Wilk | | | | | | |
|  |  | Statistic | | df | | Sig. | | | Statistic | | df | | | Sig. | |
| RoG_diff | | .084 | | 100 | | .079 | | | .982 | | 100 | | | .194 | |
| a. Lilliefors Significance Correction | | | | | | | | | | | | | | | |
| **Paired Samples Test** | | | | | | | | | | | | | | | |
|  | | | Paired Differences | | | | | | | | | t | df | | Sig. (2-tailed) |
|  |  |  | Mean | | Std. Deviation | | Std. Error Mean | 95% Confidence Interval of the Difference | | | |  |  |  |  |
|  |  |  |  |  |  |  |  | Lower | | Upper | |  |  |  |  |
| Pair 1 | RoG (complex) - RoG(Apo) | | .233 | | .122 | | .012 | .209 | | .257 | | 19.108 | 99 | | .000 |

**Summary of Statistical Analysis**

| **Parameter** | **Test Used** | **Mean Diff** | **Significance** | **Effect Size** | **Interpretation** |
| --- | --- | --- | --- | --- | --- |
| **RMSD (Complex − Apo)** | Wilcoxon Signed-Rank | 0.7038 | *Z* = −27.39, *p* < 0.001 | *r* = 0.87 (large) | Complex has higher RMSD → increased deviation from native conformation |
| **Rg (Complex − Apo)** | Paired t-Test | 0.2335 | *t*(99) = 19.11, *p* < 0.001 | *d* = 1.91 (large) | Complex has higher Rg → more expanded structure |
